# Gonadotropin-releasing hormone agonist protects ovarian function in young patients with ovarian malignancy undergoing platinum-based chemotherapy: A prospective study

**DOI:** 10.3389/fonc.2022.986208

**Published:** 2022-10-19

**Authors:** Ya Xie, Haoran Duan, Dong Wang, Huiqing Li, Jia Jia, Jialin Zhang, Linlin Li

**Affiliations:** ^1^ Department of Gynecology, The First Affiliated Hospital of Zhengzhou University, Zhengzhou, China; ^2^ Department of Obstetrics and Gynecology, Tangdu Hospital, Fourth Military Medical University, Xi’an, China; ^3^ Department of Gynecology, Shenzhen Maternal and Child Health Hospital, Southern Medical University, Shenzhen, China; ^4^ Department of Oncology, The First Affiliated Hospital of Zhengzhou University, Zhengzhou, China

**Keywords:** ovarian function, platinum-based chemotherapy, gonadotropin-releasing hormone agonist, ovarian malignancy, anti-Müllerian hormone (AMH), prospective study

## Abstract

**Purpose:**

We aimed to ascertain the effectiveness of gonadotropin-releasing hormone (GnRH) agonist co-therapy for the preservation of ovarian function in patients with ovarian malignancy who underwent unilateral salpingo-oophorectomy and platinum-based chemotherapy.

**Methods:**

We enrolled 158 patients with ovarian malignancy who underwent fertility preservation surgery and postoperative platinum-based chemotherapy between January 2018 and December 2020. Patients were divided into two groups based on the use of GnRH agonist (GnRHa) during chemotherapy. Two patients withdrew from the study. Laboratory tests (serum follicle-stimulating hormone [FSH], serum luteinizing hormone [LH], and serum anti-Müllerian hormone [AMH]) were performed pre-chemotherapy and one year post-chemotherapy. Data on menstruation resumption, perimenopausal symptoms (modified Kupperman Menopausal Index [KMI]), health-related quality of life (Medical Outcomes Study Short Form-36 [MOS SF-36]), and obstetric outcomes were collected.

**Results:**

One year post-chemotherapy, the serum AMH level in the GnRHa group was higher than that in the control group (P<0.001), while the serum FSH and FSH/LH levels in the GnRHa group were lower than those in the control group (P<0.001). The mean period from last chemotherapy to menstrual resumption was 3.86 and 5.78 months in the GnRHa and control groups (P<0.001), respectively. The rate of menstrual resumption post-chemotherapy was 93.5% and 82.3% in the GnRHa and control groups (P<0.05), respectively. GnRHa co-administration during chemotherapy reduced the likelihood of low AMH levels post-chemotherapy and was significant in the multivariate analysis (P<0.05). The modified KMI scores and MOS SF-36 scores were better in the GnRHa group than in the control group (both P<0.001).

**Conclusion:**

GnRHa protects ovarian function during platinum-based adjuvant chemotherapy in young patients with ovarian malignancy. This study provides a therapeutic reference for gynecologists, especially for those in economically and medically underdeveloped areas.

**Trial registration:**

Chinese Clinical Trial Registry (chiCTR1800019114; October 26, 2018; http://www.chictr.org.cn/index.aspx)

## Introduction

Ovarian malignancy is more frequent in postmenopausal women; however, it can also occur in young premenopausal women (1, 2). Due to the lack of effective early screening strategies, most cases are detected at advanced stage and need to receive platinum-based chemotherapy (3). As for young patients, fertility-sparing surgery that preserves the uterus and unilateral ovary, followed by 3–6 cycles of platinum-based adjuvant chemotherapy, has been the standard treatment. However, common platinum-based combination chemotherapy regimens used in ovarian malignancies have moderate toxicity to ovarian function, causing infertility and premature ovarian failure, which seriously affects the patients’ quality of life (4). As damage induced by chemotherapy to the remaining ovary is irreversible and progressive (5), gynecologists should be mindful of young patients with ovarian malignancy who undergo fertility-sparing surgery followed by chemotherapy, which may cause premature ovarian failure.

Young patients are often diagnosed with unilateral ovarian cancer; therefore, fertility preservation options and measures to protect ovarian function must be offered, considering the possibility of contralateral ovary damage during chemotherapy. Platinum-based multiagent chemotherapy used in ovarian malignancies, comprising etoposide, platinum, paclitaxel, and bleomycin, increases the risk of early menopause and infertility (6). The mechanism of ovarian function damage caused by chemotherapy drugs may include direct killing of germ cells in the ovary, depletion of a large number of primordial follicles in the ovary, damage to interstitial blood vessels in the ovary, and disruption of the ovarian germ stem cell nest microenvironment (7); however, the specific mechanism requires further research and verification.

Chemotherapy-induced gonadotoxicity decreases ovarian reserve. The degree of ovarian function damage caused by chemotherapy is mainly related to the chemotherapy regimen, drug cumulative dose, and cycles of chemotherapy. Serum anti-Müllerian hormone (AMH) levels can predict ovarian reserve (8). Apoptosis of primordial follicles reduces AMH levels, which activates the remaining follicles, leading to ovarian reserve burn-out (9). Among the available parameters, serum AMH level is a reliable and repeatable indicator of ovarian reserve in reflecting the function in the primary and secondary ovarian follicle stages (10, 11).

Currently, advanced methods for *in-vitro* temporary preservation of patient fertility have been established in developed areas, including cryopreservation of the oocyte, embryo, and ovarian tissue, whereas methods for *in-vitro* maturation of the oocyte are still in the experimental stages (12). However, oocyte and embryo freezing strategies cannot prevent primary ovarian insufficiency infertility, and there is a risk of tumor residue and recurrence during oocyte or embryo transplantation (12, 13). Moreover, these measures are expensive, require delays of weeks to months, and are not currently available in several countries and regions worldwide (2, 4). An alternative simple, low-cost, and convenient method is ovarian suppression, in which gonadotropin-releasing hormone agonists (GnRHa[s]) or GnRH antagonists are administered before and during chemotherapy to inhibit pituitary function and ultimately inhibit the maturation of intraovarian follicles. This protocol is based on the clinical premise that chemotherapy before puberty has not damaged ovarian function. Theoretically, inhibition of the pituitary-gonadal axis before and during chemotherapy can preserve the ovaries in an inactive state, thus making the follicles less vulnerable to chemotherapeutic drugs (10, 14).

GnRHa was first used in the treatment of patients with breast cancer to protect ovarian function from the damage caused by chemotherapy. Several randomized controlled clinical studies on hematological malignancies and breast cancer concluded that GnRHa combined with chemotherapy could improve the recovery rate of menstruation and spontaneous ovulation in premenopausal patients (13, 15). The degree of damage to ovarian function varies with different chemotherapy regimens and cycles. In addition, patients with ovarian malignancy have only one ovary post-surgery, increasing the vulnerability of ovarian function to chemotherapy. However, ovarian reserve function and fertility preservation with GnRHa in ovarian malignancy is still under investigation. The current literature and information are scarce (11, 15), and there is a lack of prospective studies, possibly due to concerns about the risk of ovarian cancer recurrence. The aim of this study was to ascertain the effectiveness of GnRHa for preserving ovarian function in patients with ovarian malignancy who underwent unilateral salpingo-oophorectomy and platinum-based chemotherapy in a prospective trial with a large sample size.

## Materials and methods

### Patients

This single-center, prospective study was conducted at the First Affiliated Hospital of Zhengzhou University. From January 2018 to December 2020, 158 patients with early ovarian malignancies who underwent fertility preservation surgery and postoperative chemotherapy at the First Affiliated Hospital of Zhengzhou University were enrolled. The diagnosis, treatment process, and case data were recorded in detail. Patients were divided into the control group (*n*=80) and the GnRHa group (*n*=78) according to the patients’ wishes (one person in each group was excluded). The patients participated voluntarily in the study and provided informed consent. The study was approved by the Institutional Review Board of the First Affiliated Hospital of Zhengzhou University (2017-KY-018; date of approval April 6, 2017) and was registered in the Chinese Clinical Trial Registry (chiCTR1800019114).

### Inclusion criteria

The inclusion criteria were as follows: patients 1) with a pathological diagnosis of ovarian malignant tumor with the tumor tissue confined to one ovary and the contralateral ovary normal on biopsy pathological examination; 2) who underwent unilateral salpingo-oophorectomy for ovarian malignancies to preserve the contralateral ovary; 3) aged <45 years; 4) with regular menstruation for ≥3 months before surgery, with no abnormalities in sex hormone or serum AMH levels; 5) without mental diseases, conscious, and compliant; 6) who needed chemotherapy according to postoperative pathological examination results with a chemotherapy regimen of bleomycin, etoposide, and cisplatin (BEP), etoposide and cisplatin (EP), or paclitaxel and carboplatin (TC); and 7) with ≥12 months of follow-up data.

### Exclusion criteria

The exclusion criteria were patients 1) with contraindications for chemotherapy; 2) who are allergic to GnRHa; 3) with a history of other malignant tumors; 4) receiving radiation therapy; 5) receiving other chemotherapy regimens; 6) with a long history of hormone drug use; and 7) who were unable to undergo the complete follow-up.

### Treatment

The treatment regimens for all enrolled patients were based on the 2018 National Comprehensive Cancer Network guidelines. Patients with ovarian malignancy underwent fertility-preserving surgery (removal of the unilateral ovarian fallopian tube meanwhile preservation of the opposite ovary and uterus). Routine chemotherapy was administered to the control group: patients with ovarian epithelial tumors received TC chemotherapy regimen, including paclitaxel 175 mg·m^-2^ plus carboplatin area under the curve (AUC) 5, *via* intravenous injection, given on day 1 of a 3-week cycle for 3–6 cycles; patients with ovarian malignant germ cell tumor received BEP or EP chemotherapy regimen, including cisplatin 30 mg·m^-2^ D1–3, etoposide 100 mg·m^-2^ D1–3, with or without bleomycin 15 mg·m^-2^ D1–2, over a 3-week cycle, for 3–4 cycles. Ovarian sex gonad stromal tumor received TC or BEP chemotherapy regimen, for 3–6 cycles. The GnRHa group was given conventional chemotherapy (chemotherapy regimen similar to the control group) combined with GnRHa treatment: 3.75 mg leuprolide acetate or goserelin 3.6 mg sustained-release implant was subcutaneously injected 7–14 days before chemotherapy, every 28 days until 2 weeks post-chemotherapy. A dose of 1500 mg of calcium was administered as a daily supplement during the use of GnRHa.

### Perimenopausal symptoms and quality of life

The modified Kupperman Menopausal Index (KMI) measures the presence and severity of menopausal symptoms. The 13 menopausal symptoms in the modified KMI include vasomotor function, paresthesia, insomnia, nervousness, melancholy, vertigo, weakness, arthralgia and myalgia, headache, palpitation, formication, urinary symptom, and vaginal dryness (16). The total modified KMI score is the sum of factors and severity of menopausal symptoms among participants in each group, with higher scores associated with more severe symptoms. Health-related quality of life (HRQoL) was investigated using the Medical Outcomes Study Short Form-36 (MOS SF-36) questionnaire (17). The MOS SF-36 consists of eight subscales measuring physical functioning (PF), role-physical (RP), bodily pain (BP), general health (GH), vitality (VT), social functioning (SF), role-emotional (RE), and mental health (MH) (18). The higher the score, the better the HRQoL in the measured area.

### Follow-up

Routine surveillance for recurrence, including serum markers, pelvic ultrasound, and abdominal computed tomography, was performed every 3 months for 2 years and semiannually thereafter. Serum follicle-stimulating hormone (FSH), luteinizing hormone (LH), and AMH levels were detected on day 2 to day 4 of menstruation 12 months post-chemotherapy. Menstrual resumption intervals were accurately recorded. Follow-up also included the outcomes of pregnancy in childbearing-willing patients in the two groups. The modified KMI and MOS SF-36 scores were used to evaluate the perimenopausal symptoms and HRQoL one year post-chemotherapy in both groups and were followed up regularly through outpatient review, telephone, and WeChat.

### Statistical methods

Data are presented as median values with ranges or as counts with percentages. Dichotomous and multitaxonomic variables were analyzed using the chi-square test, Fisher’s exact test, or continuity correction chi-square test, whereas continuous variables were compared using the Mann–Whitney U test or *t*-test. Univariate and multivariate logistic regression were used to analyze the clinical factors affecting AMH level. Statistical significance was set at P<0.05. Statistical software (SPSS 26.0, IBM Corp., Armonk, NY) was used for data analysis.

## Results

### Patient characteristics

**Figure 1:**
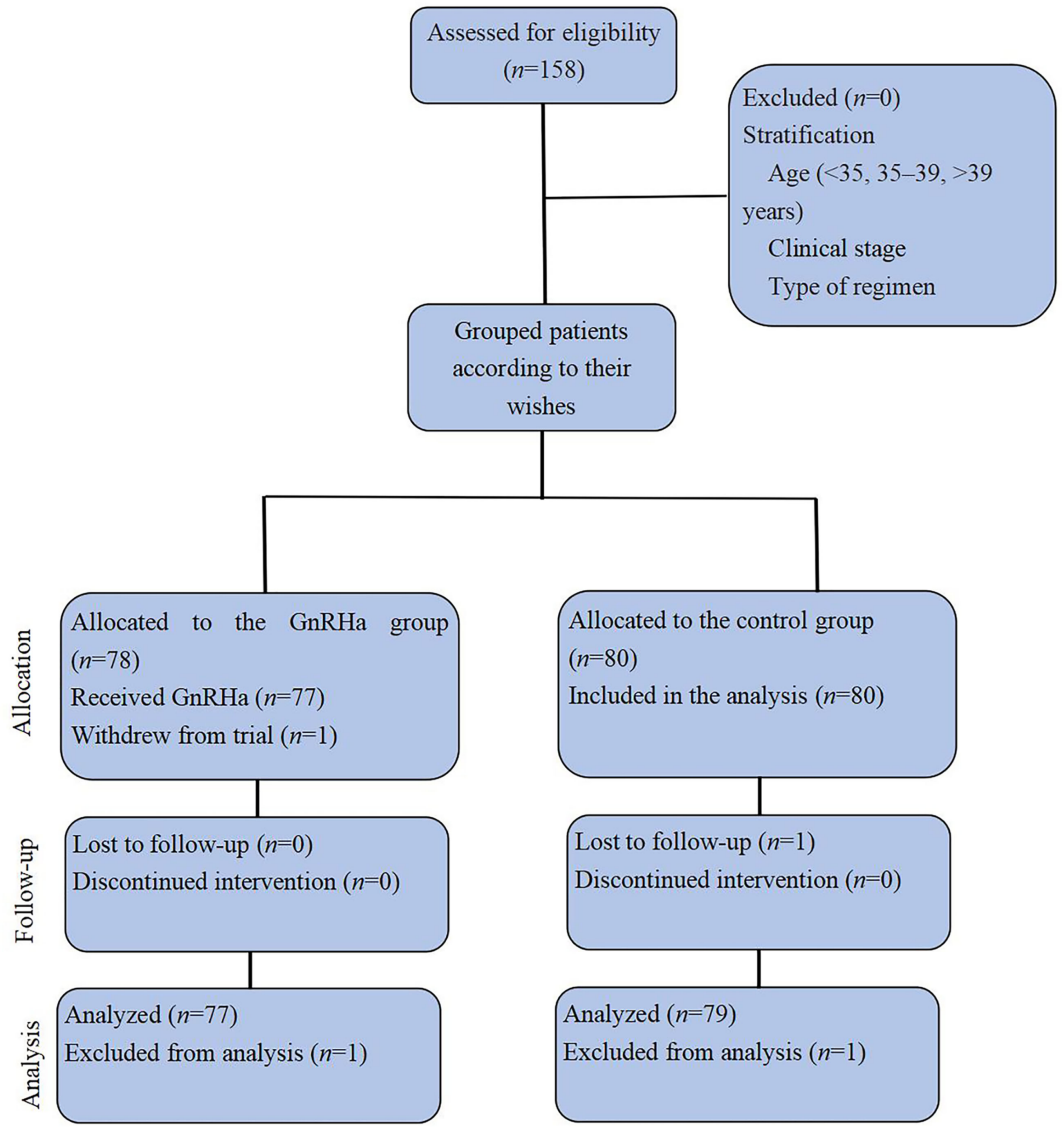
CONSORT diagram. GnRHa, gonadotropin-releasing hormone agonist.

This trial enrolled 158 patients assigned to the GnRHa (*n*=78) or control (*n*=80) groups according to the patients’ wishes (**Figure 1**). Two patients, one from each group, could not be evaluated; one patient in the GnRHa group withdrew from the trial prior to completion of the treatment and one patient in the control group was lost to follow-up. The clinical characteristics of the GnRHa (*n*=77) and control (*n*=79) groups are shown in **Table 1**. No significant differences were noted between the groups in age at diagnosis, parity, age at menarche, body mass index, level of tumor markers, clinical stage, histology type, type of surgery, chemotherapy regimens, cycles of chemotherapy, and follow-up time. The median age was 27 years (13–42 years) in the control group and 25 years (13–42 years) in the GnRHa group. No significant difference was observed in the age distribution between the two groups (P=0.173). Epithelial tumors were the most common histologic type, followed by germ cell tumors. Sex cord-stromal tumors accounted for only 10% of all tumors in the two groups. No significant difference was found in the distribution of tumor types between the GnRHa and control groups (P=0.403). The BEP/EP and TC regimens were frequently used in the GnRHa and control groups, and the distribution of chemotherapy regimens used between the two groups was not significantly different (P=0.343). The median follow-up time was 28 months from the completion of the last chemotherapy in both groups (range, 12–48 months), and there was no significant difference in the distribution of follow-up time between the two groups (P=0.835). In the GnRHa group, a median of five (range, 5−7) cycles of GnRHa were administered, with leuprolide acetate most frequently used, followed by goserelin.

**Table 1:** Patient clinical characteristics.

Characteristic	GnRHa group (*n*=77) Number, (%)	Control group (*n*=79) Number, (%)	P-value
Age, years			0.173^c^
Median (Q1, Q3)	25 (18,31)	27 (23,33)	
Range	13–42	13–42	
<35	68 (88.3)	64 (81.0)	
35–39	4 (5.2)	11 (13.9)	
40–42	5 (6.5)	4 (5.1)	
Parity			0.094^a^
Nulliparous	52 (67.5)	43 (54.4)	
Parous	25 (32.5)	36 (45.6)	
Menarche, years			0.182^d^
Median (Q1, Q3)	13 (12,14)	13 (13,14)	
BMI, kg/m^2^			0.860^e^
Median (Q1, Q3)	22.8 (21.6,23.8)	22.9 (21.6,23.9)	
Tumor markers, median (Q1,Q3)			
CA-125, U/mL	68.2 (24.7,227.9)	78.1 (36.0,430.3)	0.152^d^
CA-199, U/mL	13.9 (4.3,30.5)	12.8 (7.1,31.4)	0.631^d^
CEA, ng/ml	1.1 (0.7,2.3)	1.3 (0.7,2.1)	0.691^d^
AFP, ng/ml	2.5 (1.7,20.7)	2.9 (1.8,6.4)	0.515^d^
HE4, pmol/L	60.1 (45.5,120.6)	65.3 (51.2,110.4)	0.479^d^
Clinical stage			0.982^a^
I	44 (57.1)	45 (57.0)	
II–III	33 (42.9)	34 (43.0)	
Histology type			0.403^a^
Epithelial tumors	38 (49.4)	47 (59.5)	
Germ cell tumors	31 (40.3)	24 (30.4)	
Sex cord-stromal tumors	8 (10.4)	8 (10.1)	
Type of surgery			0.419^b^
Laparotomy	5 (6.5)	2 (2.5)	
Laparoscopy	72 (93.5)	77 (97.5)	
Chemotherapy regimen			0.343^a^
TC	44 (57.1)	51 (64.6)	
(B)EP	33 (42.9)	28 (35.4)	
Chemotherapy cycle			0.584^a^
Median (Q1, Q3)	4 (4,6)	4 (4,6)	
≤4 cycles	51 (66.2)	49 (62.0)	
>4 cycles	26 (33.8)	30 (38.0)	
Follow-up time			0.835^a^
Median (Q1, Q3)	28 (19,38)	28 (19,35)	
12–24 months	28 (36.4)	30 (38.0)	
≥24 months	49 (63.6)	49 (62.0)	
Number of GnRHa applications			
Median (Q1, Q3)	5 (5,7)		
GnRHa type			
Leuprolide acetate	52		
Goserelin acetate	25		
Recurrence	1	2	1.000^b^
Death	0	0	

GnRHa, gonadotropin-releasing hormone agonist; BMI, body mass index; CA-125, cancer antigen 125; CA-199, cancer antigen 199; CEA, carcinoembryonic antigen; AFP, alpha-fetoprotein; HE4, human epididymis protein 4; TC, paclitaxel/carboplatin chemotherapy; (B)EP, (bleomycin)/etoposide/cisplatin chemotherapy.

^a^Chi-square test; ^b^Continuity correction chi-square test; ^c^Fisher’s exact test; ^d^Mann–Whitney U test; ^e^t-test.

### Serum levels

There was no significant difference in AMH level between the GnRHa group and the control group (4.18 ± 0.96 ng/mL and 3.95 ± 0.84 ng/mL, respectively) before chemotherapy (**Table 2**). One year after chemotherapy, AMH levels decreased in both groups (P<0.001), and the GnRHa group (3.01 ± 1.06 ng/mL) had higher AMH levels than the control group (2.08 ± 0.94 ng/mL; P<0.001). The mean serum FSH level was 9.77 mIU/mL and 5.39 mIU/mL in the control and GnRHa groups (P<0.001), respectively, one year post-chemotherapy (**Table 3**). One year post-chemotherapy, the mean serum LH level was 5.80 mIU/mL in the GnRHa group and 5.89 mIU/mL in the control group, with no significant difference (P=0.918). The FSH/LH ratio in the GnRHa group was lower than that in the control group (0.98 ± 0.42 *vs*. 1.86 ± 1.01; P<0.001).

**Table 2:** AMH levels of the two groups before chemotherapy and one year after chemotherapy (x ± s).

Hormone	GnRHa group (*n*=77)	Control group (*n*=79)	t/Z	P-value
AMH, (ng/mL)				
Before chemotherapy	4.18 ± 0.96	3.95 ± 0.84	1.573	0.118^b^
One year after chemotherapy	3.01 ± 1.06	2.08 ± 0.94	5.089	<0.001^a^
t/Z	7.204	9.313		
P	<0.001^b^	<0.001^a^		

GnRHa, gonadotropin-releasing hormone agonist; AMH, anti-Müllerian hormone.

^a^Mann–Whitney U test; ^b^t-test.

**Table 3:** Hormone levels of the two groups one year after chemotherapy (x ± s).

Hormone	GnRHa group (*n*=77)	Control group (*n*=79)	P-value
FSH, (mIU/mL)			
One year after chemotherapy	5.39 ± 1.78	9.77 ± 3.49	<0.001^a^
LH, (mIU/mL)			
One year after chemotherapy	5.80 ± 1.58	5.89 ± 2.09	0.918^a^
FSH/LH			
One year after chemotherapy	0.98 ± 0.42	1.86 ± 1.01	<0.001^a^

GnRHa, gonadotropin-releasing hormone agonist; FSH, follicle-stimulating hormone; LH, luteinizing hormone.

^a^Mann–Whitney U test.

### Resumption of menses

The rate of menstrual resumption after chemotherapy was 93.5% (72 of 77) in the GnRHa group and 82.3% (65 of 79) in the control group, with a significant difference (P=0.032) between the two groups (**Table 4**). The mean interval of resumption in the GnRHa group was shorter (3.86 ± 1.44 months) than that in the control group (5.78 ± 1.27 months) (P<0.001).

**Table 4:** Comparison of menstrual recovery after chemotherapy between the two groups.

Group	*N*	Menstrual recovery time (months)	Menstrual recovery	Unrestored menstruation
GnRHa	77	3.86 ± 1.44	72	5
Control	79	5.78 ± 1.27	65	14
*X^2^/t*		8.281	4.596
P		<0.001^b^	0.032^a^

GnRHa, gonadotropin-releasing hormone agonist.

^a^Chi-square test; ^b^t-test.

### Factors affecting AMH levels one year post-chemotherapy

Results from univariate and multivariate logistic regression analyses of risk factors associated with AMH <1.1 μg·L^-1^ are presented in **Tables 5** and **6**. In the univariate analysis, parity (nulliparous *vs*. parous), clinical stage (I *vs*. II/III), body mass index, histology type (epithelial tumors *vs*. non-epithelial tumors), chemotherapy regimen (TC *vs*. [B]EP), and cycles of chemotherapy were not associated with AMH <1.1 μg·L^-1^ one year post-chemotherapy (**Table 5**). Age (odds ratio [OR], 1.093; 95% confidence interval [CI], 1.026–1.165; P=0.006), AMH levels before chemotherapy (OR, 0.535; 95% CI, 0.322–0.890; P=0.016), and GnRHa co-administration during chemotherapy (OR, 0.235; 95% CI, 0.083–0.671; P=0.007) were significant risk factors for AMH <1.1 μg·L^-1^ one year post-chemotherapy. In the multivariate analysis, GnRHa co-administration during chemotherapy was a significant factor for AMH <1.1 μg·L^-1^ one year post-chemotherapy (OR, 0.259; 95% CI, 0.088–0.760; P=0.014); however, age and AMH levels pre-chemotherapy were not significant contributors (**Table 6**).

**Table 5:** Univariate logistic regression analysis of factors contributing to AMH <1.1 μg·L^-1^ .

	AMH <1.1 μg·L^-1^		
	OR	95% CI	P-value
Age	1.093	1.026–1.165	0.006
Parity			
Nulliparous	1		
Parous	2.302	0.939-5.646	0.069
Clinical stage			
I	1		
II–III	2.348	0.948–5.813	0.065
BMI	1.308	0.988–1.731	0.060
Histology			
Epithelial tumors	1		
Non-epithelial tumors	0.736	0.298–1.818	0.507
Chemotherapy regimen			
TC	1		
(B)EP	0.640	0.247–1.660	0.359
Chemotherapy cycle			
≤4	1		
>4	0.750	0.288–1.950	0.555
AMH level before chemotherapy	0.535	0.322–0.890	0.016
GnRHa co-administration			
No	1		
Yes	0.235	0.083–0.671	0.007

AMH, anti-Müllerian hormone; BMI, body mass index; OR, odds ratio; CI, confidence interval; TC, paclitaxel/carboplatin chemotherapy; (B)EP, (bleomycin)/etoposide/carboplatin chemotherapy; GnRHa, gonadotropin-releasing hormone agonist.

**Table 6:** Multivariate logistic regression analysis of factors contributing to AMH <1.1 μg·L^-1^.

	AMH <1.1 μg·L^-1^		
	OR	95% CI	P-value
Age	1.064	0.990–1.145	0.092
AMH level before chemotherapy	0.691	0.378–1.263	0.230
GnRHa co-administration			
No	1		
Yes	0.259	0.088–0.760	0.014

AMH, anti-Müllerian hormone; GnRHa, gonadotropin-releasing hormone agonist; OR, odds ratio; CI, confidence interval.

### Disease progression

Disease progression was observed during treatment and follow-up. During follow-up (12–48 months), three patients (1.9%, 3/156) from the two groups had a relapse; however, no deaths were recorded (**Table 1**). Tumor recurrence occurred in two cases in the control group and one case in the GnRHa group. GnRHa did not affect the recurrence of ovarian tumors at the time of the last follow-up in May 2022 (P>0.05).

### Climacteric syndrome and HRQoL

**Figure 2:**
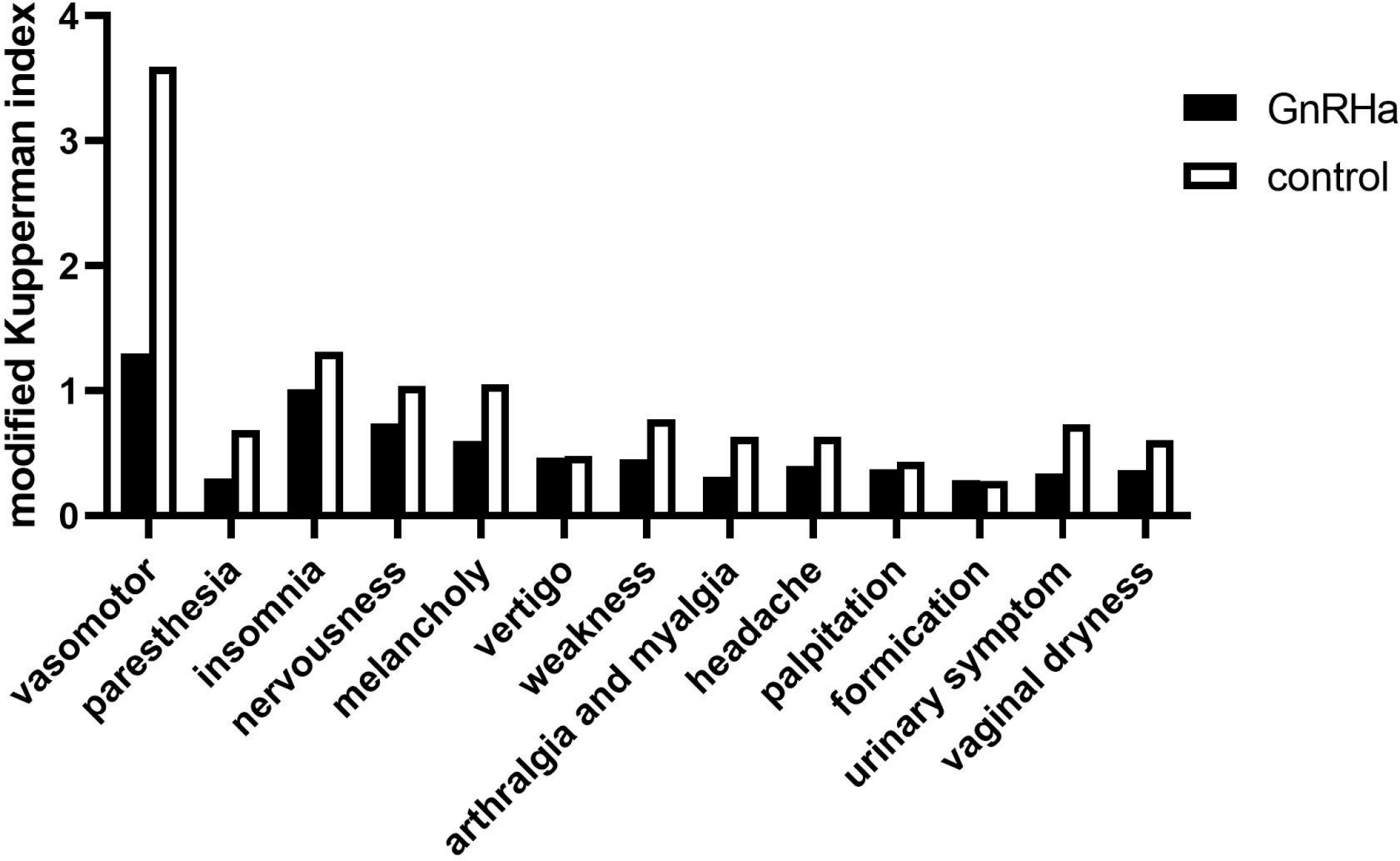
Modified Kupperman Menopausal Index of the GnRHa and control groups one year after chemotherapy. GnRHa, gonadotropin-releasing hormone agonist.

**Figure 3:**
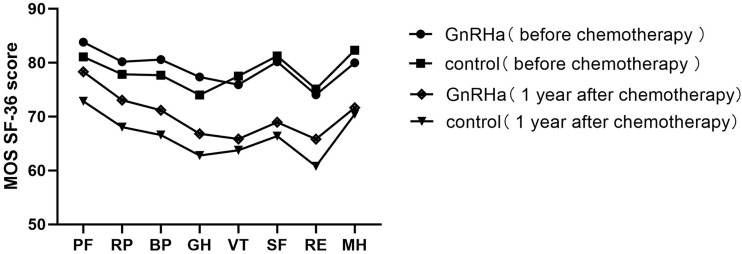
Mean MOS SF-36 subscale scores of the GnRHa and control groups before chemotherapy and one year after chemotherapy. MOS SF-36, Medical Outcomes Study Short Form-36; GnRHa, gonadotropin-releasing hormone agonist; PF, physical functioning; RP, role-physical; BP, bodily pain; GH, general health; VT, vitality; SF, social functioning; RE, role-emotional; MH, mental health.

Pre-chemotherapy, no significant difference was noted in the modified KMI and MOS SF-36 scores between the two groups (**Table 7**). The modified KMI scores of the GnRHa group and the control group one year post-chemotherapy are shown in **Figure 2**, and the MOS SF-36 scores of the two groups before chemotherapy and one year after chemotherapy are shown in **Figure 3**. One year post-chemotherapy, the modified KMI scores increased, and the MOS SF-36 scores decreased in both groups (P<0.001). The GnRHa group had a significantly lower modified KMI score and a significantly higher MOS SF-36 score than the control group (P<0.001).

**Table 7:** Comparison of the MOS SF-36 score and the modified KMI score between the two groups before chemotherapy and one year after chemotherapy.

Group	*N*	MOS SF-36 score		*Z*	P	Modified KMI score		*Z*	P
		Before chemotherapy	One year after chemotherapy			Before chemotherapy	One year after chemotherapy		
GnRHa	77	632.08 ± 41.25	561.64 ± 25.08	9.720	<0.001^a^	2.30 ± 1.91	6.95 ± 3.03	8.799	<0.001^a^
Control	79	626.89 ± 37.56	531.59 ± 51.42	9.793	<0.001^a^	2.48 ± 1.80	12.25 ± 3.84	10.852	<0.001^a^
*Z*		0.367	4.008			1.058	7.767		
P		0.714^a^	<0.001^a^			0.290^a^	<0.001^a^		

MOS SF-36, Medical Outcomes Study Short Form-36; KMI, Kupperman Menopausal Index; GnRHa, gonadotropin-releasing hormone agonist.

^a^Mann–Whitney U test.

### Obstetric outcome

We followed the obstetric outcomes of all 156 patients. Owing to the relatively young median age of the patients (25 years *vs*. 27 years), many patients did not have fertility requirements at follow-up. Of these, 26 patients attempted to conceive, and five patients (19.2%) delivered at term in the GnRHa group, whereas 23 patients attempted to conceive and 2 (8.7%) gave birth to healthy babies in the control group.

## Discussion

Patients with early ovarian malignancy tend to have an excellent prognosis after unilateral salpingo-oophorectomy followed by platinum-based chemotherapy, and most patients achieve long-term survival. However, the complications of ovarian dysfunction caused by unilateral salpingo-oophorectomy and chemotherapy, such as early menopause, infertility, perimenopausal symptoms, depression, sleep disorders, osteoporosis, and cardiovascular disease, often severely affect HRQoL in surviving patients. Moreover, premature ovarian failure is related to an increased mortality rate (19).

Previous studies have shown that serum AMH is an independent factor for predicting ovarian reserve (8, 20). In this trial, the serum AMH levels of patients decreased significantly one year post-chemotherapy, and 17.7% (14/79) of patients in the control group had amenorrhea, indicating that chemotherapy caused a certain degree of damage to the ovarian reserve function. Furthermore, to better assess the severity of perimenopausal symptoms and the impact on patients’ HRQoL caused by chemotherapy-induced ovarian dysfunction, we used the modified KMI score, a widely recognized evaluation system for perimenopausal symptoms, and the quality-of-life scale (MOS SF-36), respectively (16–18). We discovered that the modified KMI scores were worse post-chemotherapy than pre-chemotherapy in both groups (P<0.001). In addition, the MOS SF-36 scores were lower after chemotherapy in both groups (P<0.001). Thus, platinum-based combination chemotherapy used in young patients with ovarian malignancies impaired ovarian reserve function and adversely affected the patients’ HRQoL to some extent.

Our results showed that, one year post-chemotherapy, the serum FSH level and FSH/LH ratio of patients in the GnRHa group increased and were lower than those of patients in the control group, which may be associated with the function of the pituitary gland secretion and FSH suppression, as the results revealed that initial follicle recruitment and circulation were restrained and follicles were limited in the primitive follicle and presinus follicle stage (12, 21). Interestingly, one year post-chemotherapy, the serum AMH levels of patients who underwent GnRHa co-administration were higher than those in the control group (P<0.001). Moreover, GnRHa co-administration decreased the proportion of patients with AMH <1.1 μg·L^-1^ one year post-chemotherapy (P=0.014) and resulted in shorter menstrual recovery times (P<0.001) and higher menstrual recovery rates (P<0.05) than in the control group. Our results suggest that GnRHa combined with platinum-based chemotherapy can protect ovarian reserve function, thereby reducing the damage caused by chemotherapeutic drugs and reducing the risk of amenorrhea. Similarly, the MOS SF-36 and the modified KMI scores after chemotherapy were measured in our study. We found that the modified KMI and MOS SF-36 scores of patients who received GnRHa were better than those of patients in the control group (both P<0.001), suggesting that chemotherapy combined with GnRHa treatment can protect ovarian function, thus decreasing patients’ perimenopausal symptoms and improving their HRQoL.

Several previous trials in patients with breast cancer and hematologic tumors who underwent chemotherapy indicated that GnRHa co-treatment improved spontaneous resumption of menses and ovulation as a result of reducing ovarian reserve function injury (22–26); this is consistent with our results in patients with ovarian malignancy. Combination therapy with GnRHa can reduce the incidence of premature ovarian failure by inhibiting the pituitary ovarian axis, thereby reducing the number of primordial follicles entering the differentiation stage. Moreover, this therapy can act directly on the gonado-ovary and inhibit the apoptosis of follicular cells (27, 28). Concurrently, the low estrogen environment reduces blood perfusion to the ovarian tissue, resulting in a decrease in the distribution of chemotherapy drugs in the ovary. Regarding molecular mechanisms, GnRHa may promote the expression of anti-apoptotic proteins such as sphingosine 1-phosphate to protect gonadal stem cells from chemotherapeutic agents (29).

In this trial, patients were enrolled with only frequent estrogen deprivation symptoms, reversible upon discontinuation, and bone metabolism alterations not significant for therapies <6 months, which is consistent with previous studies (4, 26). To reduce the side effects of low estrogen status caused by GnRHa during chemotherapy, oral Remifemin tablets were administered. The main ingredient of Riphemin tablets is black cohosh, which is widely used to mitigate perimenopausal symptoms, such as hot flashes, night sweats, mood swings, and vaginal dryness (30). Besides, to reduce the loss of calcium, a dose of 1500 mg of calcium was administered as a daily supplement during the use of GnRHa (31). In breast cancer, GnRHa was also found to protect against chemotherapy-related ovarian function damage without significant increase in the incidence of GnRHa-related toxic effects, such as hot flashes, sweating, headache, vaginal dryness, and thromboembolic events (32, 33). Thus, the combination of GnRHa with chemotherapy is a noninvasive and less expensive method, with mild side effects, that can protect against chemotherapy-induced ovarian function damage.

Studies on GnRHa reducing chemotherapy-induced ovarian function damage in the area of ovarian cancer are rare. In a retrospective study by Zhu et al., no case of premature ovarian failure was found in 16 patients with borderline ovarian tumor and ovarian cancer treated with chemotherapy combined with GnRHa (34). Unfortunately, owing to the trial design and the small number of patients, the authors were unable to conclude that GnRHa has ovarian function protection in this patient population. In another recent retrospective study on ovarian germ cell tumors, the authors found that patients who received GnRHa combined with chemotherapy had complete menstrual recovery compared with 90.9% in a chemotherapy-alone group, thus showing a reduced risk of amenorrhea in patients who received combined GnRHa and chemotherapy (11). The results of these two studies are consistent with those of our study. A number of studies have shown that serum AMH level is not only a reliable and repeatable indicator representing ovarian reserve function, but also a sensitive and effective indicator reflecting early decline, which reflects the function of primary and secondary ovarian follicles (10, 11). In our study, serum AMH levels pre- and post-chemotherapy were accurately measured and analyzed in all patients, and our findings revealed that the ovarian reserve function was effectively protected by GnRHa. However, the AMH levels of the patients were not shown in the previously mentioned two studies (11, 34).

Consistent with previous studies, the present study revealed that the menstrual recovery rate of patients with ovarian malignant tumors with or without GnRHa after chemotherapy was 93.5% (72/79) and 82.3% (67/79), respectively, a statistically significant difference (11, 33). Moreover, the mean period of menstrual resumption in the GnRHa group was shorter than that of the control group (3.86 *vs*. 5.78 months; P<0.001), indicating that GnRHa is beneficial for the earlier menstrual recovery of postoperative chemotherapy patients. However, a retrospective study by Choi et al. showed no difference in periods of menstrual resumption. Conversely, their study showed a difference in the menstrual recovery rate (11). This discrepancy may be due to differences in the population of enrolled patients, chemotherapy regimens, and the nature of a retrospective study. The study by Choi et al. was retrospective; hence, the date of resumption of menstruation after chemotherapy relied on patients’ memories, and the reported menstrual recovery time post-treatment may have been inaccurate (11). In contrast, the patients’ menstrual recovery times were likely more accurate in our prospective study. In addition, the type and cycle of chemotherapy drugs affect the recovery of ovarian function. The chemotherapy regimens including BEP, EP, or TC exhibit moderate gonadotoxicity; however, the cyclophosphamide included in the breast chemotherapy regimen is severely toxic to ovarian function, and the cycles of chemotherapy are different. This may explain the inconsistent menstrual recovery rates and periods of menstrual resumption reported in studies of other types of cancer.

Previous studies on the effects of GnRHa on reproductive function have reported inconsistent results. In reviewing the results of 12 randomized clinical trials in breast cancer, Lambertini et al. observed that the application of chemotherapy combined with GnRHa was related to an increased chance of pregnancy (35). Two recent studies in breast cancer showed that women who received GnRHa during chemotherapy were more likely to have a pregnancy; however, because the absolute numbers were small, no significant difference was found (36, 37). In our study, the patient median age was young, follow-up time was relatively short, and several patients did not have fertility requirements during our follow-up. Further, 26 patients in the GnRHa group and 23 patients in the control group were willing to get pregnant. During the follow-up, five patients in the GnRHa group and two patients in the no GnRHa group delivered successfully.

This study has the following strengths. First, to our knowledge, this trial is the first large-sample-size prospective study to assess the protective effect of GnRHa on ovarian reserve function in patients with ovarian malignancy. Second, serum AMH levels, which can reliably indicate ovarian reserve, were measured before chemotherapy and one year after chemotherapy. In the multivariate analysis, GnRHa co-administration during chemotherapy was a significant factor for AMH <1.1μg·L^-1^ one year post-chemotherapy. To the best of our knowledge, this is also the first trial to assess ovarian reserve function by measuring serum AMH levels between GnRHa and control groups in patients with ovarian malignancy. Third, we investigated the effect of GnRHa on patients’ perimenopausal symptoms using the modified KMI scores and HRQoL using the MOS SF-36 scores, and our results suggest that GnRHa protected ovarian reserve function and improved patients’ HRQoL in early-age ovarian malignancies. In summary, in this trial, the enrolled patients were given clinically common chemotherapy regimens and cycles according to the National Comprehensive Cancer Network guideline, and the results implied that GnRHa in combination with chemotherapy may protect ovarian reserve function, reduce the risk of amenorrhea, and improve HRQoL in young patients with ovarian malignancies and that this combination does not affect tumor recurrence. Thus, for gynecologists, the results can provide a reference to assist in clinical decision-making for young patients with ovarian malignancies.

This study has some limitations. First, the patients were relatively young (median age in the GnRHa group and control group was 25 and 27 years, respectively), with a relatively short total follow-up period (12–48 months) for some patients. Therefore, some patients had no desire to procreate during the follow-up period, and the pregnancy status of the two groups could not be compared. In the future, we plan to follow up longer to establish whether GnRHa can improve fertility and long-term quality of life in patients with ovarian cancer. We will evaluate the effect of GnRHa on pregnancy, and continue to focus on hormone levels, menopausal age, HRQoL, and perimenopausal symptoms to assess the long-term impact of GnRHa combined with chemotherapy. Besides, we will evaluate the influence of GnRHa on disease recurrence and patient survival. Second, the type of ovarian malignancy and the chemotherapy regimens were not uniform in all patients. However, we ensured consistency between the two groups in the tumor types and chemotherapy regimens, leading to credible results.

According to the results of this research, platinum-based chemotherapy combined with GnRHa can effectively prevent ovarian function damage and improve HRQoL in patients with ovarian malignancy undergoing platinum-based chemotherapy. Compared with other treatments in terms of reproductive toxicity from chemotherapy, the combination of GnRHa with chemotherapy is a potential preferred treatment approach in areas limited by economic and medical technology restrictions, because this treatment approach does not affect chemotherapy regimens and has high clinical value. However, multicenter clinical trials involving large samples are required to confirm the effect of the treatment on fertility.

In conclusion, this trial is the first large-sample-size prospective trial to confirm that GnRHa protects ovarian reserve function in young patients with ovarian malignancy who underwent chemotherapy and unilateral salpingo-oophorectomy. The results of this trial can serve as a therapeutic reference for gynecologists when choosing methods to protect the ovarian reserve function in young patients, especially in economically and medically underdeveloped areas.

## Data availability statement

The original contributions presented in the study are included in the article/supplementary material. Further inquiries can be directed to the corresponding author.

## Ethics statement

The studies involving human participants were reviewed and approved by The Institutional Review Board of the First Affiliated Hospital of Zhengzhou University (2017-KY-018; date of approval April 6, 2017). Written informed consent to participate in this study was provided by the participants’ legal guardian/next of kin.

## Author contributions

YX was in charge of the project and designed and conducted the study. HD, DW, and JZ were responsible for data collection. HD and HL were responsible for the statistical analysis. The first draft of the manuscript was written by YX and HD, with suggestions from JJ, LL, and comments from all authors on previous versions of the manuscript. All authors participated in manuscript revision and approved the final version.

## Funding

This research was sponsored by the Medical Science and Technology Project of Henan Province (2018020056), the National Natural Science Foundation of China (81802770), and the Young and Middle-aged Health Science and Technology Innovation Talents Program (YXKC2020036).

## Acknowledgments

The authors wish to thank all the gynecologists at the First Affiliated Hospital of Zhengzhou University for their help in data collection. We thank all the patients that participated in this study.

## Conflict of interest

The authors declare that the research was conducted in the absence of any commercial or financial relationships that could be construed as a potential conflict of interest.

## Publisher’s note

All claims expressed in this article are solely those of the authors and do not necessarily represent those of their affiliated organizations, or those of the publisher, the editors and the reviewers. Any product that may be evaluated in this article, or claim that may be made by its manufacturer, is not guaranteed or endorsed by the publisher.

## Abbreviations

AMH, anti-Müllerian hormone; GnRH, gonadotropin-releasing hormone; GnRHa, gonadotropin-releasing hormone agonist; BEP, bleomycin, etoposide, and platinum; EP, etoposide and platinum; TC, paclitaxel and platinum; KMI, Kupperman Menopausal Index; HRQoL, health-related quality of life; MOS SF-36, Medical Outcomes Study Short Form-36; FSH, follicle-stimulating hormone; LH, luteinizing hormone; OR, odds ratio; CI, confidence interval.
